# phiBIOTICS: catalogue of therapeutic enzybiotics, relevant research studies and practical applications

**DOI:** 10.1186/1471-2180-13-53

**Published:** 2013-03-06

**Authors:** Katarina Hojckova, Matej Stano, Lubos Klucar

**Affiliations:** 1 Laboratory of Bioinformatics, Institute of Molecular Biology, Slovak Academy of Sciences, Dubravska cesta 21, Bratislava, Slovakia

**Keywords:** Enzybiotics, Database, Antimicrobial therapy, Cell wall lysis

## Abstract

**Background:**

The incidence of bacterial infections in humans along with the growing problem of antibiotic resistance is a major public health concern worldwide. Therefore it is necessary to develop novel therapeutic agents to control microbial pathogens. In this regard, enzybiotics, lytic enzymes endowed with the capacity to degrade bacterial cell wall, are a very promising group of alternative antimicrobials.

**Description:**

Numerous experimental studies have confirmed unique therapeutic capabilities of enzybiotics and hence they are worth of wider attention of the medical community. In order to summarize the state of current knowledge of enzybiotics, we have developed phiBIOTICS, an information portal about known and studied therapeutic enzybiotics. phiBIOTICS contains information on chemical and biological properties of enzybiotics together with compendium of facts retrieved from research studies, where enzybiotics were applied. Our auxiliary phiBiScan program utility is dedicated for prediction of novel potential enzybiotics.

**Conclusions:**

phiBIOTICS presents a solid body of knowledge about all studied therapeutic enzybiotics to date. The database brings high-value information on outcomes of applied research and pre-clinical trials of these prospective antimicrobial agents. This information which was scattered in research papers with heterogeneous quality and relevance is now available in the form of manually curated database. phiBIOTICS and phiBiScan are freely accessible at http://www.phibiotics.org/.

## Background

The discovery and development of antibiotics have revolutionised medicine in the 20th century. However their widespread and sometimes negligent usage led to the phenomenon of antibiotic resistance which reduced their efficiency as therapeutic agents [1]. Nowadays, diseases caused by bacterial pathogens resistant to variety of antimicrobial agents are more frequent in medical practice than just a few years ago. This issue has huge impact in terms of lives and health care expenses [2]. As the world is now facing a serious challenge in dealing with microbial threats, which were once thought to be defeated rather easily by antimicrobials, there is an urgent need for new types of antimicrobial therapy [3]. The concept of enzybiotics is very promising in this regard [4].

The term *enzybiotic* is a hybrid word from “enzyme” and “antibiotic” that has been coined to designate bacteriophage lytic enzymes endowed with the capacity to degrade bacterial cell wall and with antibacterial potential [5]. The concept of enzybiotics was subsequently shown to be wider than first though, and nowadays it refers to all enzymes that are able to cause microbial cell death (endolysins, bacteriocins, autolysins and lysozymes) and regardless of their origin (including antifungal enzymes, antimicrobial peptides and enzymes that block peptidoglycan layer synthesis) [6].

Alternative names used with respect to enzybiotics are *lytic enzymes* or *peptidoglycan hydrolases*, as enzymatic cleavage of bacterial cell wall peptidoglycan (resulting in cell lysis) represents their major mode of action. Group of peptidoglycan hydrolases consist of diverse enzymes that can be obtained from various sources. Major groups of enzybiotics include endolysins (from phages) [7,8]; autolysins and bacteriocins (produced by bacteria) [9,10]; and lysozymes (from various organisms) [11]. Amongst them, the phage endolysins held and still hold the special position as ultimate enzybiotics.

Endolysins or lysins are enzymes encoded by double-stranded DNA bacteriophages, actively produced toward the end of the phage lytic cycle to break down the bacterial cell wall for phage progeny release [12]. They target the integrity of the cell wall and attack major bonds in the peptidoglycan. Depending on their enzymatic properties, lysins fall into five major classes: (i) N-acetylmuramoyl-l-alanine amidases; (ii) endopeptidases; (iii) N-acetyl-β-d-glucosaminidase; (iv) N-acetyl-β-d-muramidases (lysozymes) and (v) lytic transglycosylases [13].

Numerous experimental studies performed *in vitro* and *in vivo* on animal models have proved enzybiotics as highly effective antibacterial agents against variety of bacterial pathogens [14]. Moreover, other important aspects of enzybiotic therapy were examined, e.g. immunogenicity of enzybiotics [15], adverse effects and emergence of resistance [8,12].

Bioinformatics is playing an important role in many aspects of drug discovery, drug assessment and drug development [16]. Biological databases covering genomic, proteomic and functional information have become significant in antimicrobial drug research. All information about representative enzybiotics and outcomes of their therapeutic application are dispersed among scientific papers and various biological databases. Recently, EnzyBase database has been published [17], collecting references and description of enzybiotics present in UniProt/Swiss-Prot database. In this paper we present phiBIOTICS, public database that collects available information about known therapeutic enzybiotics, with emphasis on relevant research studies regarding their practical application.

## Construction and content

### phiBIOTICS database

All data and information managed in phiBIOTICS were acquired manually from two main sources: (i) relevant research papers and books focused on identification and characterisation of enzybiotics and their potential use as therapeutics and (ii) public databases (UniProtKB [18], Pfam [19], BRENDA [20]). The database back-end is built upon a free and open source software bundle, where MySQL (v4.0) is used as relational database management system. The web user’s interface of the database is developed in PHP programming language (v5.2.2) according to XHTML standard (1.0 Transitional).

### phiBiScan program utility

Program module designated for search of potential enzybiotics is based on HMMER (v3.0) sequence homology search software [21] ( http://hmmer.janelia.org/), which implements probabilistic hidden Markov models profile (HMMs). The database of HMMs is compiled of 16 profiles of protein domains/families with cell wall lytic activity and families/domains associated with this activity, obtained from the Pfam database v25.0 (Pfam entry names: Glyco_hydro_25, Amidase_2, Amidase_3, Amidase_5, Peptidase_M23, Glucosaminidase, VanY, CHAP, SLT, Phage_lysozyme, Phage_lysis, LysM, Glyco_hydro_19, Hydrolase_2, Peptidase_M15_3, Peptidase_U40). The selection of these domains was preceded with an extensive literature and database search. The database is compressed and indexed with *hmmpress*. To search sequences against profile database, *hmmscan* is used with default parameters. phiBiScan program utility is written in PHP, communication with the phiBIOTICS database is facilitated via SQL statements.

## Utility and discussion

### phiBIOTICS - catalogue of therapeutic enzybiotics

We have developed phiBIOTICS, database of therapeutic enzybiotics, collecting information about all known and studied enzybiotics, relevant research studies and practical applications. Collected enzybiotics are mainly from bacteriophages, but also from other, bacterial sources. There are two basic requirements for including a new enzybiotic entry: (i) sequence has to be deposited in the UniProt database and (ii) there is publically available information about relevant research studies and/or practical applications. The database contains manually processed information about 21 enzybiotics and 69 corresponding research studies that represent currently known and studied enzybiotics.

phiBIOTICS content is accessible via simple and intuitive user’s web interface at http://www.phibiotics.org/. Results of database browsing are divided into two main sections named: *Enzybiotics Description* and *Relevant Studies*. The schematic structure of database entries is shown in Table 1. Each entry in *Enzybiotics Description* contains following items (if available): the name of the enzybiotic; recommended, systematic and alternative names (alternative names include “exact” Gene Ontology synonyms and other specific synonyms); UniProt ID; general enzybiotic's mode of action; phiBIOTICS family classification; catalysed biochemical reaction linked with information about corresponding EC number, Pfam family and attribute “Evidence” (predicted or experimental; information extracted from literature); the names and taxonomy IDs of source and target organisms. Moreover, each entry includes the list of infectious diseases caused by target organism with medical reference to related articles on Medscape eMedicine website (an online clinical medical knowledge base, http://emedicine.medscape.com), and the current state of research and applications of the particular enzybiotic. The range of available information is enhanced with numerous references to external resources and links to original papers for further reading.

**Table 1:** Schema of the phiBIOTICS database entries

**Enzybiotics description**	
*Name*	Conventional name of enzybiotic
*Recommended name*	Full name recommended by UniProt database (*submitted* or *approved*)
Systematic name*	Enzyme systematic name according to IUBMB Enzyme Nomenclature
*Alternative name*	Other name(s) in use
*UniProt ID*	Identifier of corresponding entry in UniProt database
*General mode of action*	The overall mechanism of antimicrobial action
*phiBIOTICS family*	Proposed enzybiotic family based upon enzymatic activity
*Reaction catalysed*	Biochemical reaction catalysed by the enzybiotic
*Source organism*	Name of the organism from which the enzybiotic was obtained
*Target organism*	Name of the organism(s) against which the enzybiotic is effective
*Disease*	List of diseases caused by target organisms
*State*	Current state of research and application(s)
*Reference*	Paper(s) describing enzybiotics properties
**Relevant studies**	
*Antimicrobial agent*	Name of applied enzybiotic(s) and other agents eventually
*Study type*	*in vitro* or *in vivo*
*Model*	Organism(s) used as experimental model
Administration*	Applied route of administration of the enzybiotics
*Relevant results*	Significant outcomes of the research study
Adverse effects and other issues*	Manifested side effects (e.g. toxicity, immunogenicity, health issues)
*Reference*	Paper(s) related to the study

* this item is not available for all entries.

In the section of *Relevant Studies,* information about research studies concerning enzybiotics is presented. Each entry contains the name of tested enzybiotic (in some studies in combination with other antimicrobial agent, e.g. antibiotics); type of study (*in vitro* or *in vivo*); model (organism used in a specific study); route of administration (intravenous, intranasal, etc.); relevant results (summary of achieved results); adverse effects and other aspects (including toxicity, immunogenicity, emergence of resistance, health effects and further issues affecting enzymatic activity) and reference to related research papers.

Enzymes degrading bacterial cell wall often possess different protein domain(s), but exert the same enzymatic activity. To address this, we have proposed novel classification of enzybiotics, which is based on assignment of specific enzymatic activity to individual protein domains (based on UniProt, EC classification, Pfam and Gene Ontology data). Therefore, each entry for enzybiotics is classified into one of the four proposed phiBIOTICS families. Brief characterisation of proposed enzybiotics families is summarised in Table 2.

**Table 2:** Characterisation of proposed phiBIOTICS families of enzybiotics

**phiBIOTICS family**	**Description**	**Pfam family**	**Enzybiotic(s)**
**Lysozyme**	Enzymes display lysozyme activity; hydrolyse the (1,4)-β-linkages between N-acetylmuramic acid and N-acetyl-d-glucosamine residues in a peptidoglycan and bonds between N-acetyl-d-glucosamine residues in chitodextrins.	**Glyco_hydro_25**	**Cpl-1**
			**Phage B30 lysin**
			**PlyGBS**
		CHAP*	**Phage B30 lysin**
			**PlyGBS**
**NAM amidase**	Enzymes display N-acetylmuramoyl-l-alanine amidase activity; hydrolyse the bond between N-acetylmuramoyl residues and l-amino acid residues in certain bacterial cell-wall glycopeptides.	**Amidase_2**	**LysH5**
			**LysK**
			**LytA**
			**MV-L**
			**phi11 endolysin**
			**PlyG**
			**PlyL**
		**Amidase_3**	**CD27L**
			**Ply3626**
		**Amidase_5**	**Pal**
			**PlyV12**
		CHAP*	**LysH5**
			**LysK**
			**phi11 endolysin**
**Other amidase/peptidase**	Enzymes contain CHAP (cysteine, histidine-dependent amidohydrolase/peptidase) domain. This domain has been proposed to hydrolyse γ-glutamyl containing substrates and is associated with several families of amidase domains.	**CHAP**	**PlyC**
			**Protein 17**
**Metallopeptidase**	Enzymes display metallopeptidase activity; hydrolyse the peptide bonds by a mechanism in which water acts as a nucleophile, one or two metal ions hold the water molecule in place and charged amino acid side chains are ligands for the metal ions.	**Peptidase_M23 VanY**	**LasA**
			**Lysostaphin**
			**Ply118**
			**Ply500**
			**ZooA**

* in this case CHAP domain is not responsible for the main enzymatic activity of the enzybiotic.

### phiBiScan - program utility for prediction of novel enzybiotics

**Figure 1:**
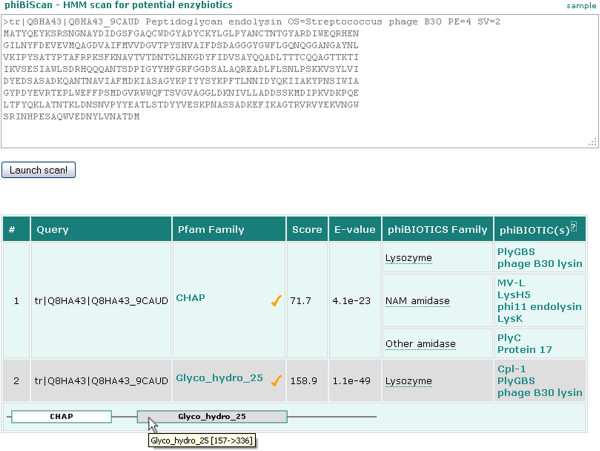
**Sample output from phiBiScan program utility.** Two domains corresponding to peptidoglycan hydrolytic activity (Pfam IDs *CHAP* and *Glyco_hydro_25*) were identified in the sequence of analysed protein.

We have developed phiBiScan, a program utility designated for prediction of novel potential enzybiotics. The program is based on sequence similarity search against hidden Markov models profiles (HMMs) of protein domains and families with lytic activity against bacterial cell wall. The phiBiScan is accessible in the *Tools* section of phiBIOTICS web portal. The input query may be single EMBL/UniProt ID or single or multiple EMBL/UniProt/FASTA entry (ies). Thus whole phage genome entries can be analysed at once. Search results are presented in tabular form. Each hit is assigned to Pfam family and to proposed phiBIOTICS family. Relevance of each hit is determined by its score and E-value. The E-value threshold is set to 1.0. Gathering threshold of a Pfam family (defined by Pfam database entry) was applied to distinguish between *significant* and *insignificant* matches (Figure 1). Position of each hit within analysed protein sequence is given in graphical form. It is important to stress the fact that phiBiScan does not provide better or more accurate results compared to standard "full" Pfam search. However, (i) it is considerably faster (especially if analysing more sequences at once), (ii) it shows only results relevant to potential enzybiotic activity and (iii) provides greater versatility for input formats.

To evaluate the overall accuracy of phiBiScan, we analysed protein sequences from known phage genomes in order to identify proteins with peptidoglycan hydrolytic activities. Phage genomes deposited in NCBI Genome database were used ( http://www.ncbi.nlm.nih.gov/sites/genome). Firstly, four groups of bacteriophages were excluded from the analysis: (i) phages lacking any peptidoglycan hydrolases, i.e. phages belonging to the families employing strategies for progeny release, which does not result in host cell lysis (*Microviridae*, *Inoviridae*, *Leviviridae*, *Lipothrixviridae*, *Rudiviridae*); (ii) unclassified phages and phages belonging to the novel phage families (e.g. *Ampullaviridae*); (iii) phages of Archaea; (iv) genomes, where no conventional peptidoglycan hydrolases were experimentally identified or predicted. Consequently the phiBiScan search was run against 37 930 protein sequences from 444 phage genomes. The number of positive and negative hits was recorded.

Going through gene annotations manually, along with additional standard Pfam search in ambiguous cases, we distinguished true and false matches. 673 proteins tested positive in phiBiScan and indeed having domain(s) corresponding to the lytic activity were considered as true positives (TP); 18 proteins tested positive, but obviously without any lytic activity were false positives (FP); 37 189 proteins tested negative and lacking lytic activity were true negatives (TN); 5 negative hits for proteins with confirmed lytic activity were considered as false negatives (FN). Solid prediction strength of phiBiScan was confirmed by high performance of binary classification test: sensitivity (99%), specificity (100%) and also positive predictive value (PPV, 97%) and negative predictive value (NPV, 100%). phiBiScan has identified 700 positive hits (567 proteins matched in one Pfam domain, 133 proteins in two Pfam domains) in 396 phages. In 48 phages no match with any applied profile was noted. Only 2 out of 18 false positive matches were assessed as *significant* positive hits, the rest were *insignificant* (Table 3).

**Table 3:** Summary of statistical assessment of phiBiScan tool

	
**True positive** (TP)	673
**False positive** (FP)	18
**True negative** (TN)	37 189
**False negative** (FN)	5
	
**Sensitivity**$\left(\frac{\mathrm{TP}}{\mathrm{TP}+\mathrm{FN}}\right)$	99%
**Specificity**$\left(\frac{\mathrm{TN}}{\mathrm{FP}+\mathrm{TN}}\right)$	100%
**PPV**$\left(\frac{\mathrm{TP}}{\mathrm{TP}+\mathrm{FP}}\right)$	97%
**NPV**$\left(\frac{\mathrm{TN}}{\mathrm{TN}+\mathrm{FN}}\right)$	100%
**Correlation coefficient**$\left(\frac{\left(\mathrm{TP}*\mathrm{TN}\right)\left(\mathrm{FP}*\mathrm{FN}\right)}{\sqrt{\left(\mathrm{TP}+\mathrm{FP}\right)\left(\mathrm{TP}+\mathrm{FN}\right)\left(\mathrm{TN}+\mathrm{FP}\right)\left(\mathrm{TN}+\mathrm{FN}\right)}}\right)$	0.98

## Conclusions

phiBIOTICS, though not an extensive collection yet, presents a solid body of knowledge about all studied therapeutic enzybiotics to date. The database brings high-value information on outcomes of applied research and pre-clinical trials of these prospective antimicrobial agents. This information which was scattered in research papers with heterogeneous quality and relevance is now available in the form of manually curated database. phiBIOTICS might be helpful for researchers examining enzybiotics, their therapeutic use and design. Curation, update and improvement process of phiBIOTICS database will be continued, with possible expansion to other areas of enzybiotics application such as agriculture or food industry.

## Availability and requirements

•Project name: phiBIOTICS

•Project home page: http://www.phibiotics.org/

•Operating system(s): Platform independent on client sides, Linux on server side

•Programming language: PHP

•Other requirements: Web browser supporting JavaScript

•License: Creative Commons Attribution-Share Alike 3.0 Unported License

•Any restrictions to use by non-academics: None

## Competing interests

All authors declare that they have no competing interest.

## Authors’ contributions

KH carried out acquisition of data for phiBIOTICS database and scoring of phiBiScan statistical evaluation, participated in conception and design of the study and drafted the manuscript. MS carried out data analysis, constructed phiBiScan utility and participated in drafting and final approval of manuscript. LK conceived of the study, participated in its design and coordination and participated in drafting and final approval of manuscript. All authors read and approved the final manuscript.
